# Antagonistic Activities of *Lactobacillus rhamnosus* JB3 Against *Helicobacter pylori* Infection Through Lipid Raft Formation

**DOI:** 10.3389/fimmu.2021.796177

**Published:** 2022-01-14

**Authors:** Anh Duy Do, Chiu-Hsian Su, Yuan-Man Hsu

**Affiliations:** ^1^ Department of Biological Science and Technology, China Medical University, Taichung, Taiwan; ^2^ Department of Animal Science and Technology, Tunghai University, Taichung, Taiwan

**Keywords:** *Helicobacter pylori*, *Lactobacillus rhamnosus*, mucus, lipid raft, Lewis x antigen, α5β1 integrin

## Abstract

*Helicobacter pylori* is a Gram-negative pathogen that can increase the risk of stomach cancer in infected patients. *H. pylori* exploits lipid rafts to infect host cells. Infection triggers clustering of Lewis x antigen (Le^x^) and integrins in lipid rafts to facilitate *H. pylori* adherence to the gastric epithelium. *H. pylori* infection can be treated with probiotics containing lactic acid bacteria that offer numerous benefits to the host while lacking the side effects associated with antibiotic therapy. Previously, we showed that the cell-free supernatant (CFS) derived from *Lactobacillus rhamnosus* JB3 (LR-JB3) at a multiplicity of infection (MOI) of 25 attenuated the pathogenicity of *H. pylori*. In this study, we established a mucin model to simulate the gastric environment and to further understand the influence of mucin on the pathogenesis of *H. pylori*. Porcine stomach mucin dramatically upregulated *H. pylori* virulence gene expression, including that of *babA, sabA*, *fucT, vacA, hp0499, cagA*, and *cagL*, as well as the adhesion and invasion ability of *H. pylori* and induced increased levels of IL-8 in infected-AGS cells. The CFS derived from LR-JB3 at a MOI of 25 reduced the expression of *H. pylori sabA, fucT*, and *hp0499* in mucin, as well as that of the Le^x^ antigen and the α5β1 integrin in AGS cells during co-cultivation. These inhibitory effects of LR-JB3 also suppressed lipid raft clustering and attenuated Lewis antigen-dependent adherence, type IV secretion system-mediated cell contact, and lipid raft-mediated entry of VacA to host cells. In conclusion, LR-JB3 could affect *H. pylori* infection through mediating lipid raft formation of the host cells. The currently unknown cues secreted from LR-JB3 are valuable not only for treating *H. pylori* infection, but also for treating diseases that are also mediated by lipid raft signaling, such as cancer and aging-associated and neurodegenerative conditions.

## Introduction

*Helicobacter pylori* is a Gram-negative pathogen that colonizes the gastric mucosa and increases the risk of stomach cancer in infected patients (1, 2). The treatment of *H. pylori* infection mainly relies on antibiotics, which may affect the balance of gut microflora and also facilitate the development of antibiotic-resistant strains (3, 4). Lactic acid bacteria are known to offer numerous benefits to the host, including protection against *H. pylori* infection (5). Previously, we indicated that oral administration of *Lactobacillus rhamnosus* JB3 (LR-JB3) to mice could eliminate *H. pylori* infection and attenuate inflammatory responses by suppressing *vacA* gene expression (6). LR-JB3 also induced superoxide dismutase and catalase activity, and the serum levels of beneficial amino acids, which are impaired by *H. pylori* infection (7). These results indicated that LR-JB3 has potential in eliminating *H. pylori* infection. Therefore, the mechanism that underlies LR-JB3 activity against *H. pylori* infection should be further investigated to support potential clinical applications.

Previously, we used a cell model to explore part of the molecular mechanism of LR-JB3 that is involved in modulating *H. pylori* infection. LR-JB3 at a multiplicity of infection (MOI) of 25 suppressed expression of *sabA* and *fucT* in *H. pylori*, translocation of CagA, and expression of the Lewis x (Le^x^) antigen and toll-like receptor 4 (TLR 4) in *H. pylori*-infected-AGS cells (8). However, it has been indicated that the Le^x^ antigen and TLR 4 are clustered in lipid rafts to facilitate the adherence of *H. pylori* to the gastric epithelial cells (9–12), and disrupting lipid rafts may prevent *H. pylori*-associated gastric cancer (13). Lipid rafts consist of phospholipids, sphingolipids, and cholesterol and act as signaling platforms and gateways for microorganisms to adhere to host cells (10, 14–17). Furthermore, *H. pylori* expresses cholesteryl α-D-glucopyranoside acyltransferase (CGAT), which is encoded by the *hp0499* gene, to enhance lipid raft clustering on host cell membranes (9). Thus, *H. pylori* can manipulate the formation of lipid rafts to promote infection. Therefore, lipid raft-mediated pathways may be involved in the mechanism of LR-JB3 against *H. pylori* infection.

The stomach is covered by a thick layer of mucus that physically protects epithelial cells from the gastric juices and interaction with pathogens (18); therefore, *H. pylori* needs to migrate through the mucus layer to colonize the epithelial cell surface (19). We used mucin to help simulate the conditions under which *H. pylori* encounters LR-JB3 in the stomach and then explored the mechanisms whereby LR-JB3 modulates *H. pylori* infection through lipid raft-mediated pathways.

## Materials and Methods

### Bacterial Strains and Cell Lines

*H. pylori* reference strain 26695 (American Type Culture Collection [ATCC] 700392) and AGS human gastric adenocarcinoma cell line (ATCC CRL-1739) were purchased from the ATCC (Manassas, VA, USA). *H. pylori* was grown on tryptic soy agar (TSA) supplemented with 5% sheep blood (Dr. Plate Biotech, Taipei, Taiwan) under microaerophilic conditions obtained using an anaerobic BD GasPak (BD Biosciences, Franklin Lakes, NJ, USA) at 37°C for 48–72 h. LR-JB3 isolated from a dairy product (6) was cultured in De Man, Rogosa, and Sharpe (MRS) broth (BD Biosciences, Franklin Lakes, NJ, USA) at 37°C for 12–18 h. AGS cells were cultured in Roswell Park Memorial Institute 1640 (RPMI) medium (Gibco-Life Technologies, Gaithersburg, MD, USA) supplemented with 10% fetal bovine serum (FBS) (Sigma-Aldrich, St. Louis, MO, USA), 100 μg/mL of penicillin, and 100 μg/mL of streptomycin (Thermo Scientific, Waltham, MA, USA) at 37°C and 5% of CO_2_. During infection experiments, AGS cells were washed three times using phosphate buffer solution (PBS), and the culture media were replaced with antibiotic-free RPMI media containing 10% FBS for further incubation.

### Mucin Preparation and Treatments of *H. pylori* and AGS Cells

Porcine stomach mucin-type II (Sigma-Aldrich, St. Louis, MO, USA) in PBS at pH 7 was sterilized by autoclaving (20). *H. pylori* harvested from TSA agar plates was resuspended at 5 × 10^6^ colony forming units (CFU) per mL with 50 mg/mL of mucin in PBS and incubated at 37°C and 5% CO_2_ for 2 h. *H. pylori* was then collected for mRNA isolation. Mucin-treated *H. pylori* was also used to infect AGS cells in the following experiments. The autoclaved mucin in PBS was centrifuged at 10,000 × *g*. Mucins were collected and resuspended in equal volumes of RPMI; 50 mg/mL of mucin in RPMI medium was then added into the culture medium for AGS cells in the control group.

### Reverse Transcription Quantitative Polymerase Chain Reaction Analysis

Total mRNA was isolated using the total RNA Miniprep Purification Kit (GMBiolab, Taichung, Taiwan), and reverse transcription was performed using the MMLV Reverse Transcription Kit (Protech Technology Enterprise, Taipei, Taiwan) according to the manufacturers’ instructions. All oligonucleotide primers used in this study were synthesized by Mission Biotech, Taipei, Taiwan. The RNA sample was incubated with random primers at 65°C for 5 min and then chilled on ice to open the secondary structure of the GC-rich RNA templates. Each sample was then incubated with a mixture of 5× Reaction Buffer, dNTP pre-mix, and MMLV reverse transcriptase. RT-qPCR was performed at the following conditions: 5 min at 65°C, 10 min at 25°C, 60 min at 42°C, and a final 10 min at 72°C. qPCR was performed in 96-well optical plates (Applied Biosystems, Waltham, MA, USA). Targeted cDNA dilutions were mixed with appropriate primers (**Table 1**) and SYBR Green Master Mix (Thermo Fisher Scientific, Waltham, MA, USA). The PCR program was: initial denaturation at 95°C for 10 min, followed by amplification for 40 cycles with denaturation at 95°C for 10 sec; annealing at 50°C for 20 sec; and extension at 72°C for 20 sec. The melting curve analysis was: 95°C for 5 sec, 65°C for 1 min, and then an increase to 95°C at 0.08°C/sec ramp rate. Each assay was run on a qPCR QuantStudio 3 (Applied Biosystems, Waltham, MA, USA) and the fold-changes in expression were derived using the comparative ΔΔC_t_ method (21). The 16S rRNA of *H. pylori* served as an internal control for sample loading and mRNA integrity (22). Results were calculated using the mean of triplicate readings.

**Table 1:** Primers used in this study.

Primer name	Sequence (5′-3′)
Mus HP16S-F	GTGTGGGAGAGGTAGGTGGA
Mus HP16S-R	TGCGTTAGCTGCATTACTGG
VacA-F	CTGCAGAAGGGAGGAAAG
VacA-R	GGCGCCATCATAAAGAGAAAT T
CagA-F	ATAATGATAAATTAGACAACTTGAGCGA
CagA-R	TTAGAATAATCAACAAACATCACGCCAT
BabA-F	TGCTCAGGGCAAGGGAATAA
BabA-R	ATCGTGGTGGTTACGCTTTTG
SabA-F	GGTGTGCTGCAACAGACTCAA
SabA-R	CATAAGCTGTTGCGCCAAATT
FucT-F	TCCAGCCCTTACTAGACGCT
FucT-R	AGCTCCAAAAGAGGGGTAGC
hp0499-F	TGTCCAATTCTTGGTATCTC
hp0499-R	ATGCGATAGGTATAGCCTAA
CagL-F	AAAACACTCGTGAAAAATACCATATC
CagL-R	TCGCTTCAAAATTGGCTTTC

### Adhesion and Invasion Assays

The adhesion and invasion abilities were defined as the numbers of *H. pylori* adhering and invading into the AGS cells, respectively. AGS cells were seeded into 24-well plates at 5 × 10^4^ cells/well in antibiotic-free RPMI medium with 10% FBS at 37°C and 5% CO_2_ for 18 h. *H. pylori* treated with mucin as previously described or PBS for 2 h was then co-incubated with mucin-treated AGS cells at a MOI of 50 at 37°C and 5% CO_2_ for 6 h. Cell culture supernatants were removed by centrifugation at 1,500 × *g* for 5 min at room temperature. The cells were then washed with PBS twice, and osmotic lysis performed to calculate the total quantity of bacteria remaining, which included adhering and invading *H. pylori*. For this purpose, sterile water was added to the infected cells following washing and the resulting cell lysates were then resuspended in PBS and plated using serial dilutions on TSA supplemented with 5% sheep blood. These plates were then cultured with 100 μL from each dilution at 37°C for 48 h. Bacterial cell numbers were determined by manual colony counting. To determine the number of viable intracellular bacteria, the standard gentamicin assay was applied (23). The same batch of infected cells was washed three times in PBS and incubated with 100 μg/mL of the membrane-impermeable antibiotic gentamicin (Sigma-Aldrich; Merck Millipore, Darmstadt, Germany) at 37°C and 5% CO_2_ for 1.5 h to remove extracellular bacteria (23). Bacterial cell numbers were then determined by manual colony counting. The adherent bacteria number was calculated by deducting the invading bacteria number from the total sum. The adhesion and invasion activities of *H. pylori* was determined as the mean of triplicate readings at each treatment. AGS cells incubated with 50 mg/mL of mucin in RPMI medium at 37°C and 5% CO_2_ for 6 h were assigned as the control group, whereas AGS cells infected by PBS-pretreated and mucin-pretreated *H. pylori* were assigned as the non-mucin group and the mucin treatment group, respectively. The non-mucin group was used to establish 100% adhesion and invasion. The cell-free supernatants (CFSs) were used in the detection of interleukin (IL)-8.

### Enzyme-Linked Immunosorbent Assay of IL-8 Levels

Detection of IL-8 in the supernatants of *H. pylori*-infected human AGS cells was conducted using human IL-8 ELISA Ready- SET-Go!R ^®^ kits (eBioscience, Inc., San Diego, CA, USA) according to manuals’ instruction. Each sample was analyzed individually. Results were calculated as the mean of triplicate readings.

### Pretreatment of *H. pylori* or AGS Cells Using CFS for Co-Cultivation

CFSs were collected from the cultures grown with 5 × 10^4^ cell/mL of AGS cells, 5 × 10^6^ CFU/mL of *H. pylori*, 1.25 × 10^6^ CFU/mL of LR-JB3, and 5 × 10^6^ CFU/mL of LR-JB3 for 2 h and were referred to as AGS, HP-100, JB3-25, and JB3-100, respectively. CFSs were mixed with 100 mg/mL of porcine stomach mucin at a 1:1 ratio for the pretreatment study. For the *H. pylori* pretreatment group, CFSs with mucin were co-incubated with *H. pylori* at 37°C for 2 h. The pretreated *H. pylori* was collected by 10,000 × *g* for 1 min at room temperature and then resuspended in RPMI medium. These *H. pylori* at a MOI of 50 were used to infect AGS cells in an antibiotic-free RPMI medium supplemented with 10% FBS at 37°C in 5% of CO_2_. For the AGS cell pretreatment group, these cells were co-incubated using CFSs with mucin at 37°C and 5% of CO_2_ for 2 h and then infected by mucin-pretreated *H. pylori* at a MOI of 50. After 6 h of incubation, the culture supernatants of both pretreatment groups were then used for the detection of IL-8 levels *via* ELISA. *H. pylori* in co-culturing conditions were assessed *via* adhesion and invasion assays. The total mRNA was isolated for the virulence gene expression in *H. pylori*. AGS cells co-incubated with 50 mg/mL of mucin in RPMI medium at 37°C and 5% of CO_2_ for 6 h were assigned as control group. AGS cells infected by mucin-pretreated *H. pylori* were referred to as the infection group. Results were calculated as the mean of triplicate readings.

### Preparation of Cell Extracts and Western Blot Analysis

Cells or bacteria were lysed with ice-cold RIPA buffer (Genestar Biotechnology, Kaohsiung, Taiwan). Protein concentration was determined using the Bradford method (Bio-Rad, Hercules, CA, USA). Protein sample (30–40 µg) was separated *via* SDS-PAGE using a Hoefer mini VE system (Amersham Biosciences, Piscataway, NJ, USA). Proteins were transferred to Immobilon-E polyvinylidene fluoride membrane (Merck Millipore, Carrigtwohill, County Cork, Ireland) according to the manufacturer’s instructions. Following the transfer, the membrane was washed with Tris-Buffered Saline (TBS) and blocked for 1 h at 37°C with 5% fat-free milk in TBS and 0.1% of Tween-20 (TBST). Diluted primary antibodies were added and co-incubated with the membrane at 4°C overnight. Blots were then incubated with peroxidase-conjugated secondary antibodies horseradish peroxidase-conjugated goat anti-mouse IgG or goat anti-rabbit IgG. Following removal of the secondary antibody, blots were washed with TBST and developed using the ECL-western blotting system (Advansta, San Jose, CA, USA). Immunoblot densities were quantified using the Hansor Luminescence Imaging System LIS02 (Han-Shuo Life Technology, Taichung, Taiwan).

### Immunofluorescence Microscopy

Twelve-millimeter diameter glasses (Paul-Marienfeld GmbH & Co. KG, Am Wöllerspfad, Lauda-Königshofen, Germany) were coated with 300 µL collagen purified from calfskin (Sigma-Aldrich, St. Louis, MO, USA) and placed on the bottom of a 24-well plate for 2 h. *H. pylori* and AGS cells were co-cultivated as described in the previous section. After 6 h of cultivation, the supernatant was removed and AGS cells were washed three times with PBS. AGS cells were then fixed with 4% paraformaldehyde for 20 min. Triton X-100 (1%) (Thermo Fisher Scientific, Waltham, MA, USA) was added and incubated for 20 min. The cells were then washed three times with PBS and co-incubated with Alexa Fluor 594-conjugated cholera toxin subunit β for GM1 (lipid rafts marker) staining for 1 h, or with the primary antibody for integrin α5 (1:1,000), β1 (1:500), or sialyl Le^x^ antibody (1:50) at 4°C for 16 h. Visualization of integrins and Lewis antigens was applied using fluorescently tagged secondary antibodies (Alexa Fluor 488, green). AGS cells were then washed three times with PBS, and 4,6-diamidino-2-phenylindole (DAPI, Southern Biotech, Birmingham, USA) was added and the mixture incubated for 5 min at room temperature. Samples were imaged at a magnification of 40× using a Revole Immunofluorescence microscopy (Echo, San Diego, CA, USA).

### Antibodies

The primary antibodies used for Western blot analysis were as follows: rat anti-integrin β1 clone AIIB2 (Merck, MABT409), rabbit anti-integrin α5 clone 5E18 ZooMAb (Sigma-Aldrich, ZRB1122-25UL), mouse anti-CD15s (sialyl Lewis^x^) (Santa Cruz, sc-32243), mouse anti-CagA (Santa Cruz, sc-28368), mouse anti-CagA phospho (Santa Cruz, sc-508), mouse anti-VacA (Santa Cruz, sc-32746), rabbit anti-p38 MAPK (Agrigo, ARG55258), rabbit anti-p38 MAPK phospho (Agrigo, ARG51850) rabbit anti-Cox-2 (Agrigo, ARG56421), goat anti-ATF-2 (Arigo, ARG63122), rabbit anti-ATF2 phospho (Agrigo, ARG42620), mouse anti-GAPDH (Agrigo, ARG10112). Furthermore, the secondary antibodies were as follows: donkey anti-goat IgG antibody (Arigo, ARG65352), goat anti-rabbit IgG (pre-adsorbed to 10 nm gold) (Abcam, ab27234), goat anti-rabbit IgG H&L (HRP) (Merck Millipore, AP132P), goat anti-mouse IgG H&L cross-adsorbed secondary antibody (Alexa Fluor 488) (Thermo Fisher, A-11001), goat anti-mouse IgG (HRP) (Biosciences, 554002) Goat anti-Rat IgG (HRP) (Thermo Fisher, 31470), goat anti-rabbit (Alexa Fluor 488) (Invitrogen A11034), goat anti-rat IgG H&L cross-adsorbed secondary antibody (Alexa Fluor 488) (Thermo Fisher, A-11006), and goat anti-mouse IgM CFL488 (Santa cruz, sc-395784).

### Statistical Analysis

Data were analyzed using SAS 9.4 software (SAS, Inc., Cary, NC, USA). Statistical significance was assessed between two groups using Student’s *t* test and Dunnett. Results were presented as the mean ± standard error of the mean. *p*<0.05 was considered to indicate statistical significance.

## Results

### Effects of Mucin on *H. pylori* Virulence Gene Expression

**Figure 1:**
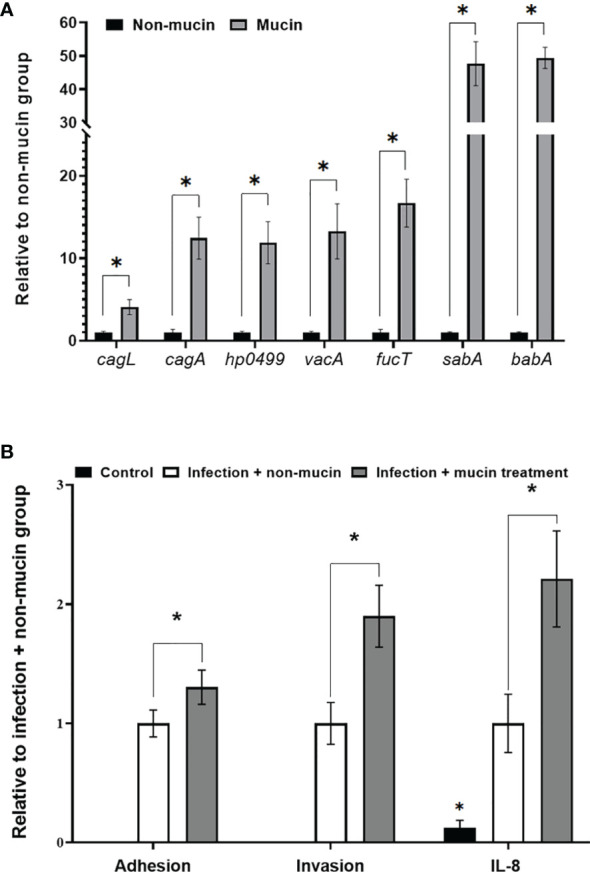
Effects of porcine stomach mucin on **(A)** virulence gene expression of *H. pylori*, and **(B)** the adhesion and invasion abilities of *H. pylori* to AGS cells and IL-8 levels of *H. pylori*-infected-AGS cells. *H. pylori* (5 × 10^6^ CFU/mL) was treated with PBS (non-mucin group) or in 50 mg/mL mucin (pH 7) at 37°C and 5% CO_2_ for 2 h (mucin group). After cultivation, total mRNAs were isolated and assessed *via* RT-qPCR to measure the *H. pylori* virulence gene expression. PBS- or mucin-pretreated *H. pylori* at a MOI of 50 were co-cultivated with AGS cells at 37°C and 5% CO_2_ for 6 h AGS cells incubated with 50 mg/mL of mucin in RPMI medium for 6 h were assigned as the control group. The number of *H. pylori* adhering and invading into AGS cells was calculated by colony counting, and the IL-8 levels in the supernatant were measured by ELISA. *Indicates significant differences compared with the non-mucin group, p < 0.05.

After entering the lumen of the stomach, *H. pylori* moves through the mucus layer to increase the accessibility of adhering to gastric epithelial cells (24). Therefore, we investigated the effects of mucin on *H. pylori* virulence gene expression in this study. The mRNA expression of *sabA* and *babA* increased nearly 50-fold compared with that of the non-mucin group (**Figure 1A**) while the expression of *cagL*, *cagA*, *vacA*, and *fucT* was upregulated from 4.0- to 17.7-fold. Furthermore, mucin-pretreated *H. pylori* showed stronger adherence and invasion abilities to AGS cells than the untreated bacteria (**Figure 1B**). These results indicated that mucin enhanced the virulence of *H. pylori*.

### Effects of CFSs With Mucin on *H. pylori* Virulence Gene Expression

**Figure 2:**
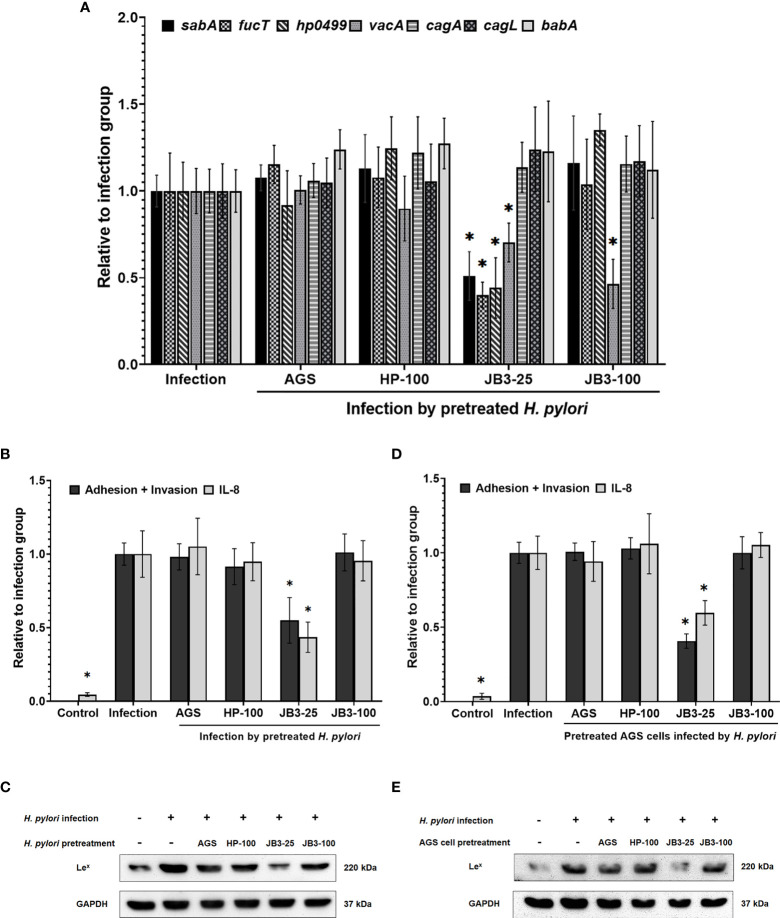
The effects of *H. pylori* **(A–C)** or AGS cells **(D, E)** pretreated using CFSs with mucin on **(A)** the mRNA expression of *sabA*, *fucT*, *hp0499*, *vacA*, *cagA*, *cagL*, and *babA* of *H. pylori* and **(B, D)** the adhesion and invasion abilities of *H. pylori*, IL-8 levels and **(C, E)** Le^x^ antigen of *H. pylori*-infected-AGS cells after 6 h co-cultivation. CFSs were collected from AGS cells at 5 × 10^4^ cell/mL, *H. pylori* at 5 × 10^6^ CFU/mL, LR-JB3 at 1.25 × 10^6^ CFU/mL, and LR-JB3 at 5 × 10^6^ CFU/mL growing in RPMI medium were referred to as AGS, HP-100, JB3-25, and JB3-100 respectively. CFSs were mixed with 100 mg/mL of porcine stomach mucin at 1:1 ratio for pretreating *H. pylori* or AGS cells. After pretreatment, *H. pylori* and AGS cells were co-cultivated at 37°C and 5% CO_2_ for 6 h. The AGS cells incubated with 50 mg/mL of mucin in RPMI medium for 6 h were assigned as the control group. The AGS cells infected by mucin-pretreated *H. pylori* were assigned to the infection group. The number of *H. pylori* adhering and invading into AGS cells was calculated by colony counting, and the IL-8 levels in the supernatant were measured by ELISA. Total proteins isolated from cell lysis were used for Western blot analysis. * indicates statistically significant differences compared with the infection + non-mucin group, p < 0.05.

The inhibitory effect LR-JB3-derived CFSs at a MOI of 25 (JB3-25) or 100 (JB3-100) on *H. pylori* infecting AGS cells through the secretion of unknown cues has been previously demonstrated, including suppressing the expression of *sabA*, *vacA*, and *fucT* in *H. pylori*, and the *H. pylori*-induced Le^x^ antigen levels in AGS cells (8). Therefore, to study the effects of mucin on this phenomenon, *H. pylori* or AGS cells were treated using different CFSs with mucin before co-cultivation. Pretreating *H. pylori* JB3-25 with mucin suppressed expression of *sabA*, *vacA*, and *fucT* mRNAs (**Figure 2A**), while expression of *vacA* mRNA was further decreased by pretreatment with JB3-100 with mucin. The adhesion and invasion abilities of *H. pylori* to AGS cells as well as *H. pylori-*induced IL-8 levels were also attenuated by pretreating *H. pylori* or AGS cells using JB3-25 with mucin (**Figures 2B, D**). Furthermore, the Le^x^ antigen expression in *H. pylori* infected-AGS cells was decreased in both pretreatment groups using JB3-25 with mucin (**Figures 2C, E**). This indicated that the inhibitory effects of JB3-25 remained in the presence of mucin which significantly increased the virulence of *H. pylori*. Interestingly, pretreating *H. pylori* using JB3-25 with mucin was able to affect the Le^x^ antigen expression in AGS cells.

### The Effects of CFSs in Mucin on *H. pylori*–Induced Lipid Raft Clustering of AGS Cells

**Figure 3:**
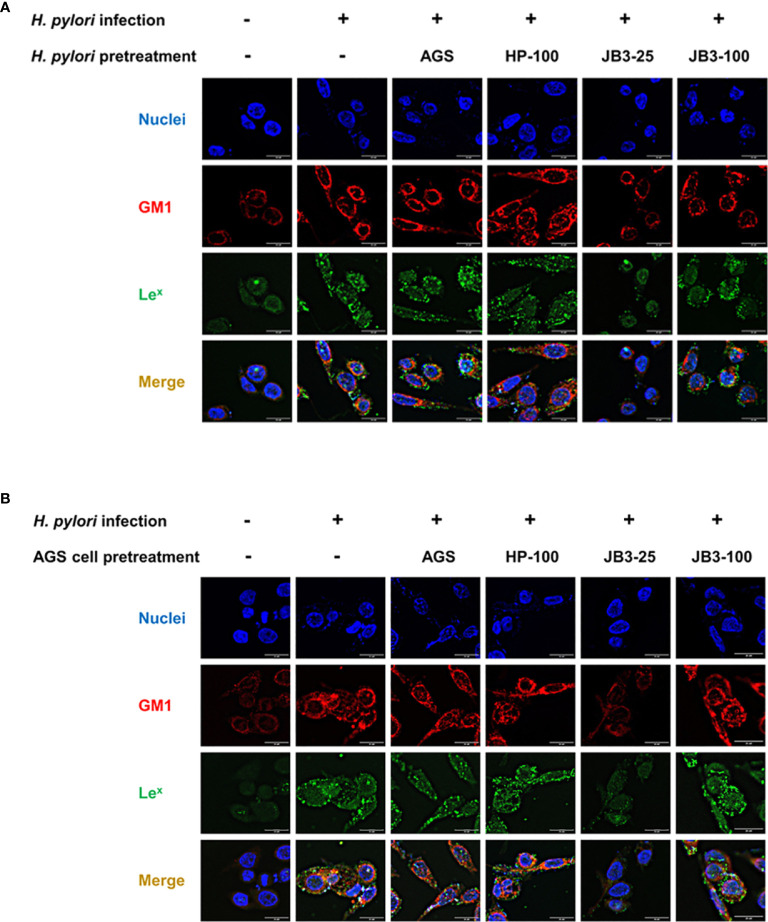
Representative fluorescence microscopy images demonstrated the effect of **(A)** *H. pylori* or **(B)** AGS cells pretreated using CFSs with mucin on the lipid rafts clustering (red) and Le^x^ antigen (green) gathering in the rafts domain in AGS cells after 6 h co-cultivation. CFSs were collected from AGS cells at 5 × 10^4^ cell/mL, *H. pylori* at 5 × 10^6^ CFU/mL, LR-JB3 at 1.25 × 10^6^ CFU/mL, and LR-JB3 at 5 × 10^6^ CFU/mL growing in RPMI medium were referred to as AGS, HP-100, JB3-25, and JB3-100 respectively. CFSs were mixed with 100 mg/mL of porcine stomach mucin at 1:1 ratio for pretreating *H. pylori* or AGS cells. After pretreatment, *H. pylori* and AGS cells were co-cultivated at 37°C and 5% CO_2_ for 6 h The AGS cells incubated with 50 mg/mL of mucin in RPMI medium for 6 h were assigned as the control group. The AGS cells infected by mucin-pretreated *H. pylori* were assigned to the infection group. Samples were imaged at a magnification of 40×.

To observe the formation of lipid rafts on the *H. pylori*-infected-AGS cells, GM1 was used as a marker to locate lipid rafts (25). Le^x^ antigen clustered in the lipid raft after *H. pylori* infection (**Figures 3A, B**). In both pretreatment groups, the lipid raft clustering as well as Le^x^ antigen co-localization were reduced by JB3-25 with mucin. Mucin treatment also increased the levels of *hp0499* mRNA expression by 11.9-fold compared with those treated in the non-mucin group (**Figure 1A**), whereas pretreatment using JB3-25 with mucin suppressed *hp0499* mRNA expression to the same degree as that of *fucT* and *sabA* (**Figure 2A**).

**Figure 4:**
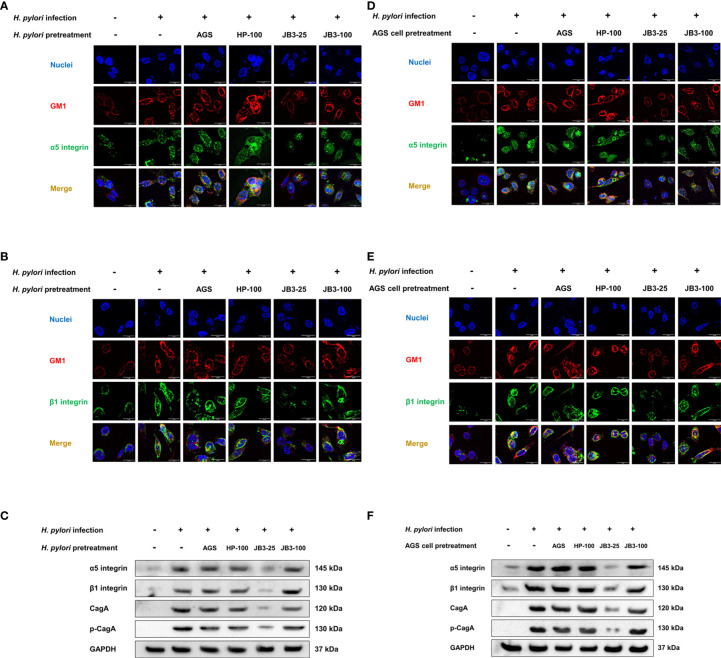
Representative fluorescence microscopy images and Western blot analysis of the effects of *H. pylori* **(A–C)** or AGS cells pretreated **(D–F)** using CFSs with mucin on the lipid rafts clustering (red), **(A, C)** α5 integrin (green), and **(B, D)** β1 integrin (green) gathering in the rafts domain, and **(C, F)** the expression of α5 integrin, β1 integrin, translocated CagA, and phosphorylated CagA in AGS cells after 6 h co-cultivation. CFSs were collected from AGS cells at 5 × 10^4^ cell/mL, *H. pylori* at 5 × 10^6^ CFU/mL, LR-JB3 at 1.25 × 10^6^ CFU/mL, and LR-JB3 at 5 × 10^6^ CFU/mL growing in RPMI medium were referred to as AGS, HP-100, JB3-25, and JB3-100 respectively. CFSs were mixed with 100 mg/mL of porcine stomach mucin at 1:1 ratio for pretreating *H. pylori* or AGS cells. After pretreatment, *H. pylori* and AGS cells were co-cultivated at 37°C and 5% CO_2_ for 6 h. The AGS cells incubated with 50 mg/mL of mucin in RPMI medium for 6 h were assigned as the control group. The AGS cells infected by mucin-pretreated *H. pylori* were assigned to the infection group. Samples were imaged at a magnification of 40×.

CagA is a major virulence factor of *H. pylori* that is delivered into host cells *via* the type IV secretion system (T4SS). CagL is a pilus component of T4SS and interacts with α5β1 integrin to trigger the translocation of CagA into host cells (10, 11). Translocated CagA is then phosphorylated and activates nuclear factor-kappa B (NF-κB) leading to the expression of proinflammatory cytokine IL-8 (26, 27). The expression of both *cagA* and *cagL* in *H. pylori* were induced by mucin but were not affected by JB3-25 with mucin (**Figures 1A**, **2A**). However, the level of *H. pylori*-induced-IL-8 in AGS cells was suppressed by mucin pretreated with JB3-25 in both pretreatment groups (**Figure 2B**). Therefore, we then studied the association of α5β1 integrin with lipid rafts. After pretreating of *H. pylori* or AGS cells by JB3-25 with mucin, both lipid raft clustering and α5β1 integrin co-localization were suppressed (**Figures 4A, B, D, E**). The expression of α5β1 integrin (**Figures 4C, F**) along with the amounts of translocated CagA and phosphorylated CagA (**Figures 4C, F**) were all reduced in both pretreatment groups.

**Figure 5:**
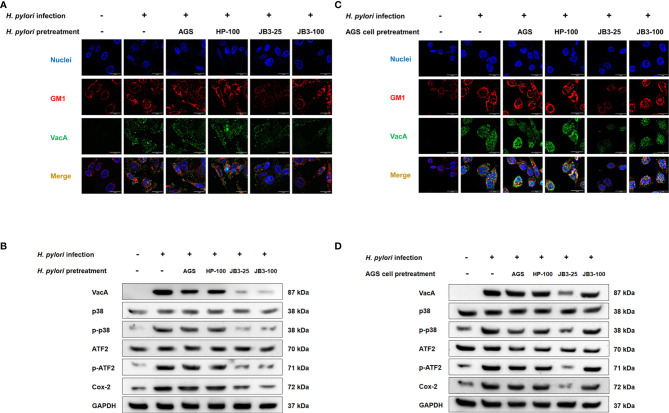
Representative fluorescence microscopy images and Western blot analysis demonstrated the effects of *H. pylori* **(A, B)** or AGS cells pretreated **(C, D)** using CFSs with mucin on the lipid rafts clustering (red), **(A, C)** intracellular VacA (green) gathering in the rafts domain, and **(B, D)** the expression of p38, phosphorylated p38, ATF-2, phosphorylated ATF-2, and Cox-2 in AGS cells after 6 h co-cultivation. CFSs were collected from AGS cells at 5 × 10^4^ cell/mL, *H. pylori* at 5 × 10^6^ CFU/mL, LR-JB3 at 1.25 × 10^6^ CFU/mL, and LR-JB3 at 5 × 10^6^ CFU/mL growing in RPMI medium were referred to as AGS, HP-100, JB3-25, and JB3-100 respectively. CFSs were mixed with 100 mg/mL of porcine stomach mucin at 1:1 ratio for pretreating *H. pylori* or AGS cells. After pretreatment, *H. pylori* and AGS cells were co-cultivated at 37°C and 5% CO_2_ for 6 h The AGS cells incubated with 50 mg/mL of mucin in RPMI medium for 6 h were assigned as the control group. The AGS cells infected by mucin-pretreated *H. pylori* were assigned to the infection group. Samples were imaged at a magnification of 40×.

VacA is a multifunctional toxin that causes vacuolation, apoptosis, and autophagy and that also regulates cell adhesion and inflammatory responses (28). VacA has been demonstrated to be internalized into host cells through an interaction with fatty acid chains of phospholipids, glycosphingolipids, and sphingolipids enriched in lipid raft domains (29–31), which triggers the p38/ATF-2-mediated signaling pathway and induces inflammatory responses (32). Here, VacA was co-localized within the lipid raft after *H. pylori* infection (**Figures 5A, C**). In the *H. pylori* pretreatment group, both pretreatment using either JB3-25 or JB3-100 with mucin reduced the amount of VacA localizing in the lipid raft domain, whereas pretreatment using JB3-100 with mucin did not affect the clustering of lipid rafts (**Figure 5A**). The levels of p-p38, p-ATF2, and Cox-2 triggered by translocated VacA in *H. pylori* infected-AGS cells were also decreased by the same pattern (**Figure 5B**). However, pretreated AGS cells using JB3-100 with mucin did not suppress VacA delivery into the AGS cells, and the levels of p-p38, p-ATF2 and Cox-2 proteins were therefore maintained compared with those in the infection group (**Figures 5C, D**).

## Discussion

The current gold standard in treating *H. pylori* infection is triple therapy combining a proton pump inhibitor and two antibiotics (33). However, antibiotic therapy has limitations including development of antibiotic-resistant strains (34) and disruption of the balance in intestinal microflora, which can lead to gastrointestinal diseases (35). Considering these side effects of antibiotic therapy, a new therapeutic strategy for *H. pylori* infection is urgently required. We have previously shown that LR-JB3 has potential in attenuating *H. pylori* infection *in vivo* and *in vitro* (6–8). Therefore, in this study, we further investigated a number of the underlying mechanisms.

Mucus plays a key role as a physical barrier that protects epithelial cells from the acidic gastric juice of the stomach and also from the entrance of pathogens (36). Studies have indicated that mucin promotes the expression of virulence genes in *Pseudomonas aeruginosa* and *Clostridium septicum* (37, 38); however, most of the cell-based models do not include mucus in the study. Furthermore, *Lactobacilli* has been shown to colonize in the mucus layer of the stomach where *H. pylori* expresses urease to reduce mucus viscosity to facilitate movement toward the gastric epithelial cells (19, 39). The mucus layer is the location where LR-JB3 and *H. pylori* first encounter each other in the stomach. Therefore, mucins were used in our cell model to offer an alternative view of the interaction between LR-JB3 and *H. pylori.*

In this study, the presence of mucin dramatically increased the expression of *sabA* and *babA* in *H. pylori*. These genes both encode outer membrane proteins that are involved in the adherence of *H. pylori* to highly glycosylated mucins *via* binding to the Lewis b (Le^b^) antigen and sialyl-Lewis x (SLe^x^) antigen, respectively (40). FucT is an enzyme involved in the Le^x^ biosynthesis of lipopolysaccharide (LPS) O-antigen of *H. pylori* (2) and *fucT* mRNA expression was also significantly increased by the mucin. Thus, our data suggests that mucin treatment facilitated *H. pylori* adhering to gastric epithelial cells in a Lewis antigen-dependent manner. Furthermore, the expression of two well-known virulent factors in *H. pylori*, *cagA* and *vacA*, was also increased. These virulence factors affect various host cell pathways such as cell inflammatory response, vacuolation, and apoptosis (41). Thus, passing through the mucus layer of the stomach increased the ability of *H. pylori* to infect gastric epithelial cells. However, LR-JB3 inhibited *H. pylori* colonization, which is agreement with our previous findings (8), even though the virulence of *H. pylori* was boosted by mucin.

Interestingly, the infection of JB3-25-pretreated *H. pylori* could also suppress the expression of infection-induced α5β1 integrin and Le^x^ antigen in AGS cells. *H. pylori* infection is known to trigger the expression of α5β1integrin to enhance CagA translocation, thereby promoting gastric pathogenesis (42). Furthermore, *H. pylori* infection has been shown to stimulate the CagA-dependent expression of β3 GlcNAc T5 (β3GnT5), which is a GlcNAc transferase essential for the expression of Lewis antigens on the epithelial cell surface (43). Therefore, JB3-25 may regulate the cellular responses of AGS cells through modulating the virulence of *H. pylori*. The unknown cue in JB3-25 suppressed the ability of *H. pylori* to induce the expression of α5β1integrin in AGS cells, sequentially reduce the amount of the translocated CagA, and then downregulate the expression of Lewis antigens in AGS cells.

α5β1 integrin and Le^x^ antigen are gathered in the raft domain to promote the adhesion of *H. pylori* to epithelial cells and initiate pathogenicity (9). The lipid raft plays an important role in the process of *H. pylori* infection. Pretreating AGS cells by JB3-25 reduced lipid raft clustering, and pretreating *H. pylori* also decreased its expression of CGAT, which participates in the lipid raft formation of the host cells. An inhibitor of CGAT, amiodarone, could prevent *H. pylori* from adhering to AGS cells and effectively suppressed the translocation of CagA into AGS cells (9). Therefore, the unknown cue in JB3-25 may contain CGAT inhibitor-like molecules to interfere with the gathering of the lipid raft and further attenuating *H. pylori* infection.

In our previous study, the levels of 15 amino acid levels were decreased in *H. pylori*-infected mice (7). Among these, phenylalanine and proline are able to reduce cellular cholesterol levels through repressing the *ABCA1* gene that mediates cholesterol synthesis (44). LR-JB3 treatments could restore plasma levels of phenylalanine and proline in infected mice. A cholesterol synthesis inhibitor simvastatin could also reduce CagA translocation *via* disrupting lipid raft clustering (13). β-cyclodextrin has been reported as an agent that disrupts cholesterol and sphingolipid-rich microdomains to facilitate the disassembly of lipid rafts leading to reduce VacA internalization into Hela cells (45). Thus, the unknown cue in JB3-25 may disrupt the formation of lipid rafts through regulating the cholesterol levels in host cells.

In contrast to JB3-25, JB3-100 specifically reduced the expression of the *vacA* gene. This result is consistent with our previous finding (8). Here, mucin treatment increased *vacA* gene expression by 13-fold compared with that in the non-mucin group, although the inhibitory effect of LR-JB3 on *vacA* expression was still maintained. JB3-100 pretreatment did not affect infection-induced lipid raft formation and the expression of α5β1 integrin and Le^x^ antigen in pretreated AGS cells. Thus, JB3-100 only acted on *H. pylori* but not on host cells. The major difference between JB3-25 and JB3-100 are the densities of LR-JB3 in the cultures. This finding implies that bacterial density plays an important role in producing metabolites that interact with both *H. pylori* and AGS cells.

**Figure 6:**
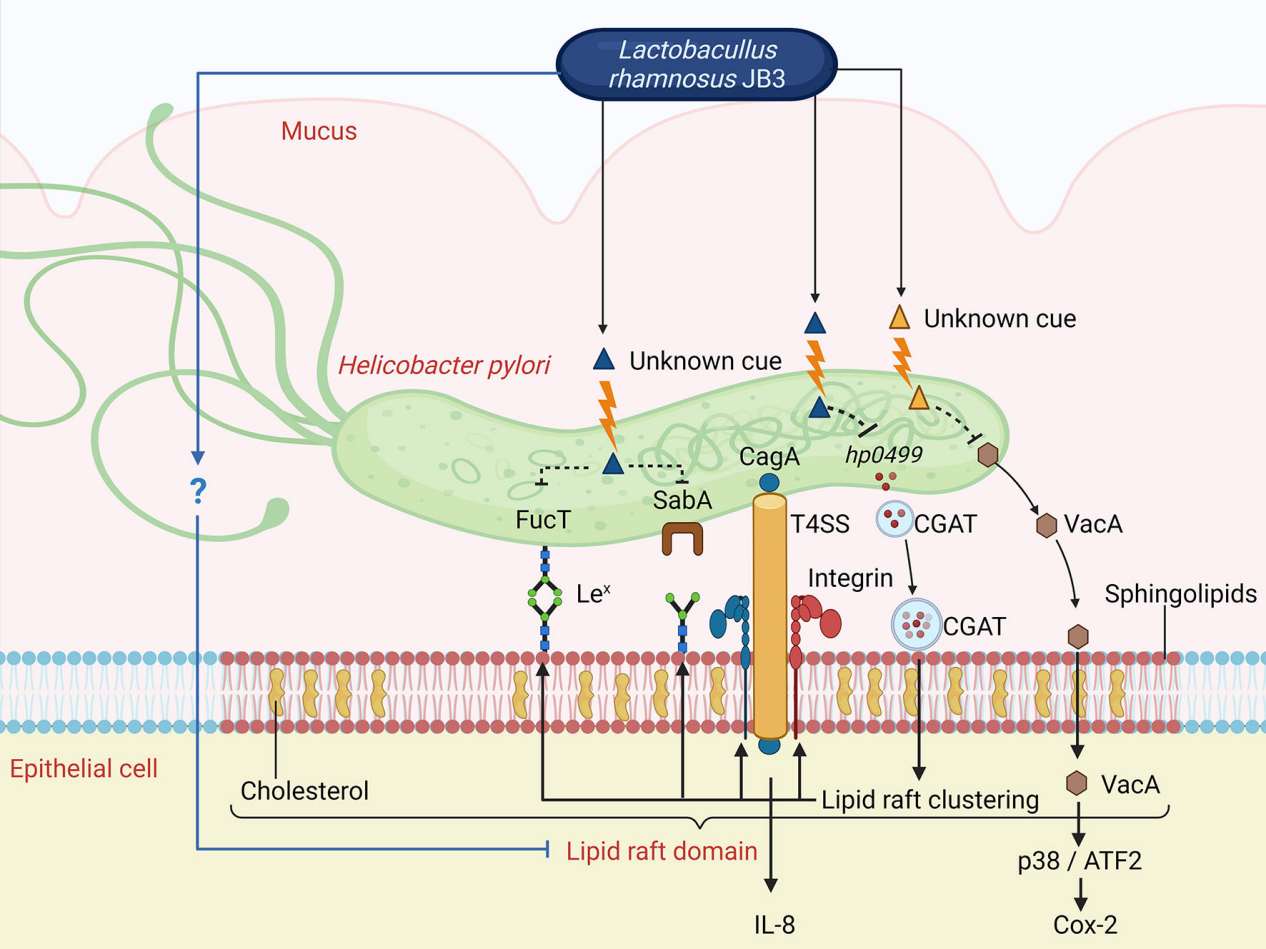
The possible pathways of CFSs derived from LR-JB3 against *H. pylori* infection (for further details see in the text). Le^x^, Lewis x antigen; T4SS, type IV secretion system; ATF2, Activating transcription factor 2; CGAT, Cholesteryl α-D-glucoside 6-acyltransferase. ​The figure was created with BioRender.com.

A summary of the possible pathways of CFSs derived from LR-JB3 against *H. pylori* is shown in **Figure 6**. Briefly, *H. pylori* infection triggers the formation of lipid rafts and recruitment of the Le^x^ antigen and α5β1 integrin to the lipid raft domains to facilitate the adherence of *H. pylori* to epithelial cells; CGAT is also secreted by *H. pylori* and is delivered to epithelial cells to enhance lipid rafts clustering. *H. pylori* expresses SabA and FucT to increase Lewis antigen-dependent adherence. CagL the adhesion subunit of T4SS binds to α5β1 integrin for T4SS-dependent CagA delivery causing the expression of IL-8. T4SS also enhances the adherence of *H. pylori* to AGS cells. Furthermore, the translocated VacA is also gathered in the lipid raft and can stimulate the p38/ATF-2-mediated signal pathway to induce inflammatory responses. The unknown cue derived from JB3-25 (blue triangle) can interfere with lipid raft formation, reducing CGAT expression in *H. pylori*, and suppressing the expressions of the Le^x^ antigen and α5β1 integrin in AGS cells. Therefore, Lewis antigen-dependent adherence, T4SS-mediated cell contact, and lipid raft-mediated entry of VacA are all attenuated. Furthermore, another unknown cue (yellow triangle) from JB3-25 and JB3-100 inhibits the internalization of VacA resulting in a reduced Cox-2 level mediated by the p38/ATF-2-mediated signal pathway. The reduced intracellular levels of VacA are caused by suppressed expression in *H. pylori* using LR-JB3 treatment. The mechanism of unknown cues from LR-JB3 that interfere with the lipid raft clustering on *H. pylori*-infected-AGS cells requires further investigation.

To the best of our knowledge, this is the first study to show that *L. rhamnosus* can affect *H. pylori* infection through mediating lipid raft formation of the host cells. The unknown cues secreted from LR-JB3 are valuable not only for treating *H. pylori* infection but also for treating diseases mediated by lipid raft signaling, such as cancers and neurodegenerative and aging-associated diseases (46).

## Data Availability Statement

The original contributions presented in the study are included in the article/supplementary material. Further inquiries can be directed to the corresponding author.

## Author Contributions

Y-MH designed this study. ADD and C-HS performed experiments; Y-MH and ADD wrote the paper. All authors approved this final manuscript. All authors have read and agreed to the published version of the manuscript.

## Funding

This research was funded by Ministry of Science and Technology (Taiwan) grant number MOST109-2320-B-039-009-MY3 and China Medical University Hospital grant number DMR-108-141.

## Conflict of Interest

The authors declare that the research was conducted in the absence of any commercial or financial relationships that could be construed as a potential conflict of interest.

## Publisher’s Note

All claims expressed in this article are solely those of the authors and do not necessarily represent those of their affiliated organizations, or those of the publisher, the editors and the reviewers. Any product that may be evaluated in this article, or claim that may be made by its manufacturer, is not guaranteed or endorsed by the publisher.
